# Clinical Manifestations of Severe Untreated Hypothyroidism

**DOI:** 10.7759/cureus.26595

**Published:** 2022-07-05

**Authors:** Abhishek S Bhutada, Thomas V Kodankandath

**Affiliations:** 1 Neurosurgery and Neurology, Virginia Tech Carilion School of Medicine, Roanoke, USA; 2 Neurology, Carilion Clinic, Roanoke, USA

**Keywords:** hypothyroid, hypothyroid-related dysphonia, hypothyroid pericardial effusion, hypothyroid patients, endocrine disorders, hypothyroid myxedema coma

## Abstract

Thyroid hormones play a crucial role in maintaining homeostasis throughout the human body. Hypothyroidism is a result of insufficient circulating levels of thyroid hormone. In a hypothyroid state, not only do all metabolic processes tend to slow down but so do neurological processes. Here, we present an interesting case of a woman with a history of severe hypothyroidism that was untreated for the past 10 years.

## Introduction

Numerous vital organ systems are regulated by thyroid hormones. Thus, thyroid deficiency is associated with a broad spectrum of symptoms implicating most body functions. Due to the insidious nature of hypothyroidism, most patients often do not seek medical treatment until late into the disease course. Currently, symptomatic hypothyroidism affects nearly 0.3% of adults in the United States (12 years of age or older) and subclinical hypothyroidism affects nearly 4.3%. It is more common in females, especially older with lower body mass index, due to the prevalence of autoimmunity in this population [1].

The case we have outlined here depicts some of the pathognomonic clinical manifestations of severe hypothyroidism that were left untreated for many years. We offer critical insights on diagnostic measures of hypothyroidism, the pathophysiology of some of the complications associated with hypothyroidism, and the importance of careful management of this disease process.

## Case presentation

A 59-year-old female was brought by her coworker to the emergency department (ED) with an episode of presyncope. She had been well until two years ago when she gradually started noting malaise, lethargy, hoarse voice, slowed speech, edema of the face and extremities, and progressive weight gain. She had not seen a doctor for the past 10 years. Her past medical history was significant for chronic neck pain after a motor vehicle accident. Family history was significant for hyperthyroidism in her mother and sister.

**Figure 1:**
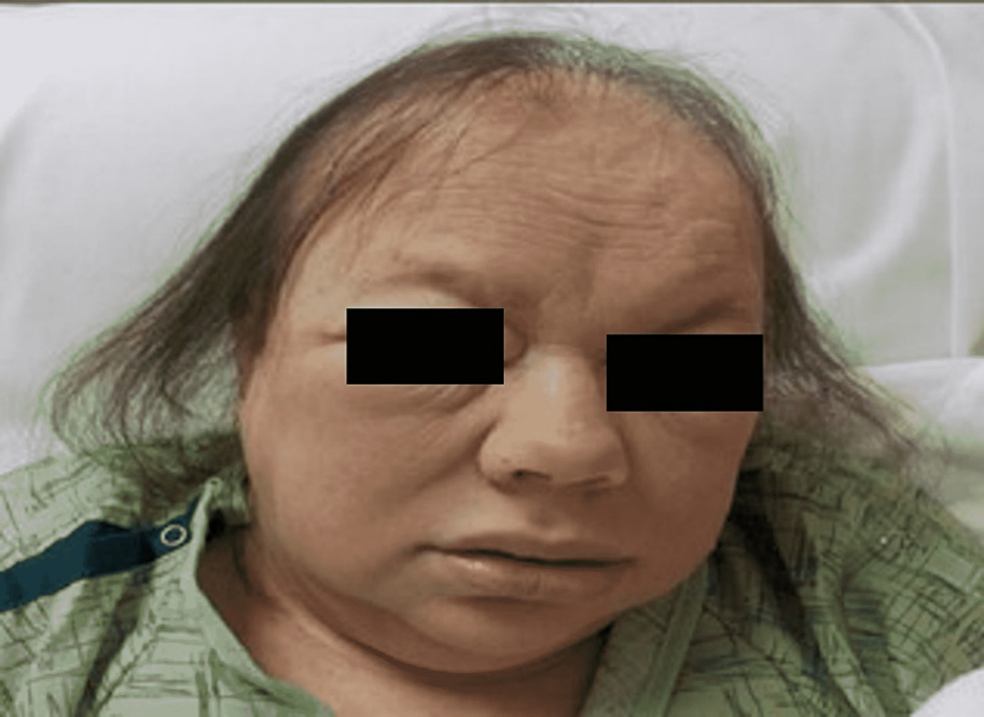
Picture of the patient highlighting physical features of baldness, loss of eyebrows, and myxedematous facies.

**Figure 2:**
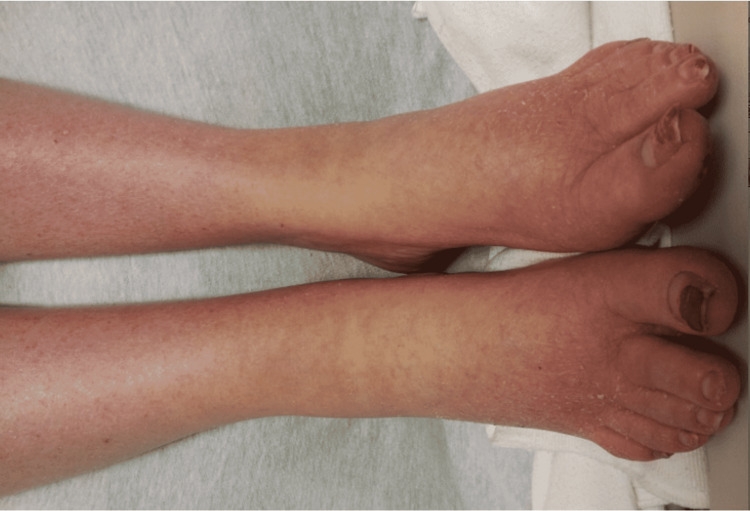
The patient’s extremities revealed dry skin and non-pitting edema.

Vital signs on admission were recorded (temperature of 93.1°F, blood pressure of 102/69 mmHg, heart rate regular at 56 beats/minute, and respiratory rate at 12 breaths/minute). Physical examination revealed a bald, pale, obese woman who spoke in a croaking voice. The head and neck examination was significant for baldness, loss of eyebrows, and myxedematous facies (Figure 1). There was no goiter, and neck veins were not distended. The cardiac apical impulse was not visible and palpable. The heart sounds were soft and distant, with no murmurs, gallops, or rubs. The abdomen exam was significant for mild hepatomegaly and was otherwise unremarkable. Extremities showed dry skin and non-pitting edema (Figure 2). Neurologic examination showed bilateral upper and lower extremities motor strength of 4/5 proximally and 5/5 distally. Bilateral grasp reflexes and prolongation of the recovery phase of the tendon reflexes were also noted on the exam.

**Figure 3:**
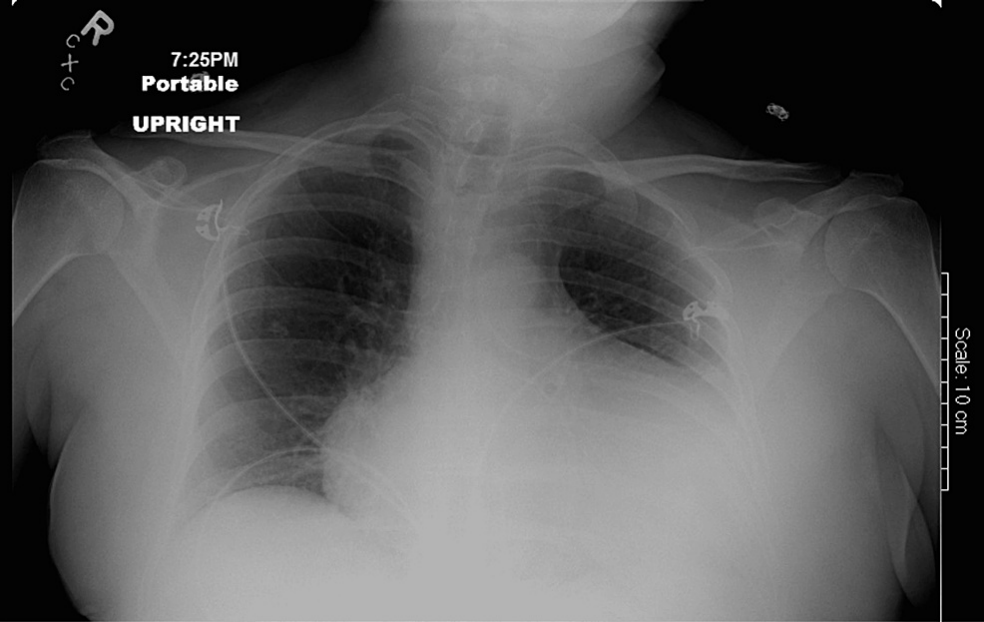
Chest X-ray revealed a classic “water bottle” appearance of the cardiac silhouette suggestive of pericardial effusion.

**Figure 4:**
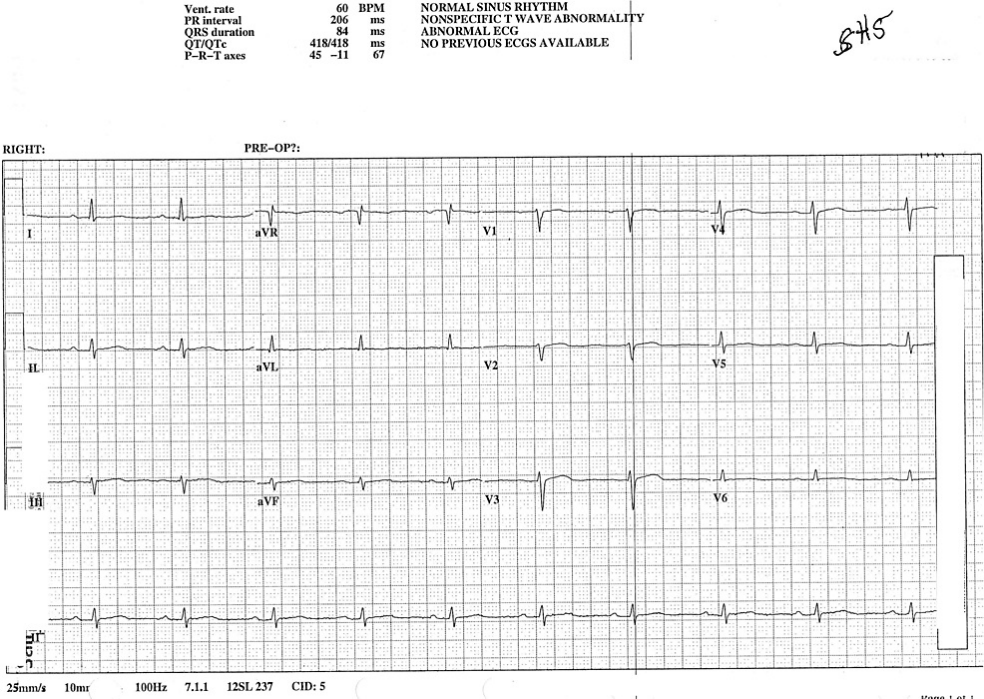
The patient's electrocardiogram revealed a heart rate of 60 beats per minute with a low-voltage pattern and first-degree atrioventricular block.

**Figure 5:**
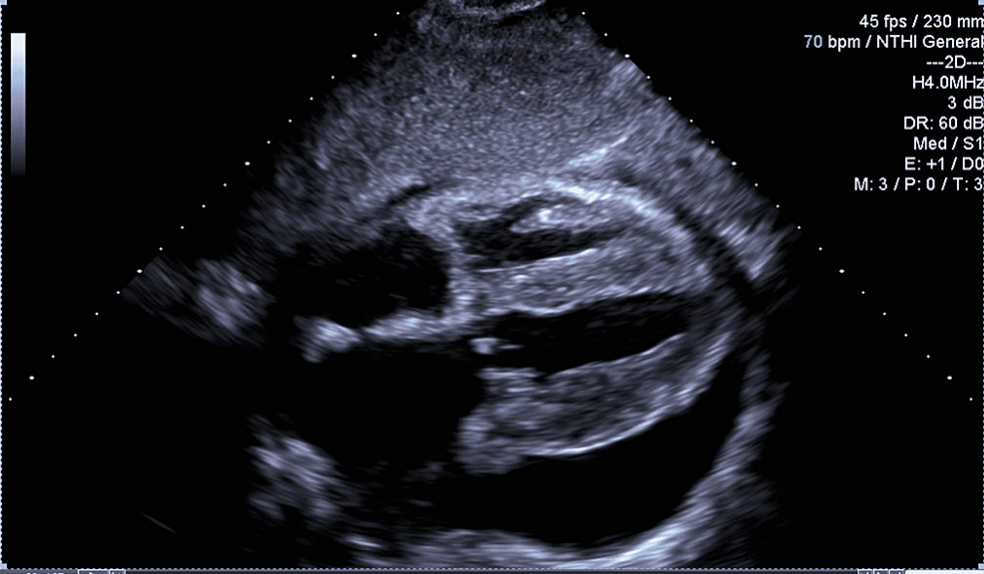
A bedside echocardiogram revealed massive pericardial effusion.

A chest radiogram showed cardiomegaly with globular enlargement of the cardiac silhouette with a “water bottle” configuration (Figure 3). An electrocardiogram showed a heart rate of 60/minute with a low voltage pattern and first-degree atrioventricular block. There was no evidence of electrical alternans or ST-T wave changes (Figure 4). A bedside echocardiogram demonstrated a small heart size with massive pericardial effusion (Figure 5). There was a swinging motion of the heart, within large effusion; however, there was no evidence of right ventricular and right atrial collapse during the diastole. Pericardiocentesis was not done, given there was no evidence of tamponade clinically and in the echocardiogram.

Laboratory values were also obtained for diagnostic purposes. A complete blood count (CBC) showed leukopenia and anemia. A thyroid function test revealed her ultrasensitive thyroid-stimulating hormone (TSH) was greater than 100 Uiu/ml and free T4 was 0 ng/dl. The afternoon cortisol level was 12.2 mcg/dL. The patient's creatinine phosphokinase was 3288 units. Liver function tests were significant for aspartate aminotransferase (AST) and alanine aminotransferase (ALT) of 196 u/L and 145 u/L, respectively, with alkaline phosphatase and total bilirubin within normal limits. Thyroglobulin and thyroperoxidase antibodies were negative. The etiology of hypothyroidism was unclear but suspected to be underlying thyroiditis possibly due to infectious causes.

The patient was started on levothyroxine 1.6 mcg/kg after adrenal insufficiency was ruled out. The patient's stay was complicated by acute hypercapnic respiratory failure and was placed on bi-level positive pressure and improved hypercarbia within a day. The patient was discharged home with levothyroxine. She was followed up in the medical clinic three weeks later and her thyroid function tests were TSH of 2.29 mIU/ml and free T4 of 0.85 ng/dL.

## Discussion

Hypothyroidism is one of the most prevalent endocrine disorders commonly affecting females. If left untreated, it can lead to severe complications for the patient. Diagnosing a person with hypothyroidism can be challenging due to its insidious onset and nonspecific findings. It can be especially challenging in patients with atypical clinical findings and can be easily overlooked in elderly patients with multiple underlying conditions. Therefore, it is necessary to obtain a thorough patient history and careful physical examination to appropriately diagnose patients with hypothyroidism.

Some characteristic appearance seen in these patients includes dry, coarse, and brittle head and body hair, which can result in total or partial alopecia. Loss of hair from the lateral third of the eyebrow is also commonly seen in these patients. The rate of hair growth is also slow. This could be due to the reflex vasoconstriction caused by a decrease in metabolic rate [2]. Hypothermia, resulting from decreased metabolic rate, is commonly seen in patients with low thyroid hormones.

Another complication seen in these patients is myxedema. The dermal accumulation of mucopolysaccharides, especially hyaluronic acid and chondroitin sulfate, results in a myxedematous state. These patients often present with nonpitting edema due to these molecules' ability to bind water. Myxedema gives the characteristic appearance of periorbital puffiness, thick lips, acral swelling, and an enlarged tongue [2]. Thyroid hormone replacement has shown to reverse this myxedematous state slowly, but it is important for these patients to comply with therapy, as the rate of recurrence is quite high.

Patients with hypothyroidism can often have respiratory complications presenting with dyspnea, airway obstruction, sleep-disordered breathing, decreased response to chemical stimuli, hypercapnia, and respiratory failure. Respiratory muscle weakness is directly proportional to the degree of thyroid dysfunction [3].

The cardiovascular system is also affected by thyroid hormones. Specifically, decreases in thyroid hormone will decrease heart rate and contractility, resulting in lower cardiac output. Decreased cardiac output leads to dyspnea on exertion in patients with low levels of thyroid hormone. Thyroid hormone regulates genes that encode myocardial enzymes responsible for the contraction and relaxation of the heart [4,5].

As seen in our patient, pericardial effusion affects nearly one-third of patients with hypothyroidism [6]. It is dependent on the severity of the disease and usually manifests during the advanced severe stages [7]. Increased permeability of the capillaries causes leakage of fluid high in protein into the interstitial space. The rate at which fluid accumulates is slow, which is why cardiac tamponade is an extremely rare complication in patients with hypothyroidism [8-10]. Since thyroid replacement has shown to effectively correct the effusion, pericardiocentesis is not necessary for these patients, unless tamponade is present.

## Conclusions

Our patient presented with many of the common features of severe hypothyroidism including malaise, lethargy, hoarse voice, partial alopecia, loss of eyebrows, myxedematous facies, and pericardial effusion. As a clinician, any of the above-mentioned features in a patient should indicate concerns of hypothyroidism. A careful history, physical exam, and appropriate labs can help establish a diagnosis earlier, prevent delay in treatment, and attain a better prognosis for the patient.
